# A Peculiar and Interesting Injury

**Published:** 1899-07

**Authors:** E. P. Flower

**Affiliations:** Senior Student, U. S. College of Veterinary Surgeons


### A PECULIAR AND INTERESTING INJURY.

By E. P. Flower,
SENIOR STUDENT, U. S. COLLEGE OF VETERINARY SURGEONS.

About February 1st a bay mare was brought into the hospital suffering with marked dyspnœa, caused by the presence of a hard fibrous-feeling enlargement midway down the neck, and which seemed to have originated behind the trachea, distorting the anatomy of the parts as it had developed, and deviating the trachea from its normal position toward the left. The history elicited from her owner was to the effect that she had been kicked while out at pasture some three months since. Shortly after the infliction of the injury a slight enlargement was discovered which increased in size as time progressed. After thorough examination and palpation of the parts a probable fracture of several of the cartilaginous rings with the secondary formation of a fibroma was diagnosed.

The animal being extremely spirited and somewhat fractious, her efforts in attempting resistance to examination, and repeated efforts to secure her, exaggerated the dyspnœic condition to such an extent that she became almost frantic in the effort to perform respiration. After the elapse of probably fifteen or twenty minutes, during which time the patient had been removed to a large, quiet box-stall, the distressing symptoms, to a great extent, became alleviated. Tracheotomy was then quietly performed immediately below the enlargement, when, to the surgeon's surprise, a double polypus was found filling up the lumen of the tube—one, suspended from the membrane above, becoming distinctly constricted as it neared the middle of the trachea, the other originating from the inferior portion of this constriction by a slender pedicle, flat in shape and swung like a pendulum, oscillating with each inspiration and expiration, and thus being the etiological explanation of the extremely difficult breathing when the animal became excited. The écraseur was passed around this abnormal growth at its superior portion, just at its junction with the membrane of the trachea and the growths extracted. The fingers were then inserted, and worked around the outside of the trachea, coming in contact with some foreign substance firmly imbedded in the muscular parenchyma, the projection which had backed up against the trachea, thus causing the deviation in position, and producing the intense inflammation of which these polypi were the product. After enlarging the above incision sufficiently to permit the introduction of a pair of heavy forceps, this object was grasped with them, and extracted with vigorous traction and some difficulty, whereupon it proved to be a fragment of a chestnut rail, about one-inch in diameter and five inches in length, frayed and sharp upon the point, and exceedingly well preserved. Four times were the forceps again inserted, and in each instance their withdrawal was attended with as many fragments, ranging in proportion from one-half to three inches in length. These splinters were found to be encysted in a degenerated capsule of a caseous nature, probably the organized inflammatory exudates. The most remarkable feature attending the presence of these foreign substances was the fact that the morbid alterations produced were comparatively small, especially as their entrance had been effected with violence, attended with laceration at the point of entrance, and at no time had there been any suppuration. The owner of the animal finally remembered that in May, 1898, while at pasture she had forcibly collided with the fence during her antics, producing a small wound at side of neck which thoroughly healed in a few days, and which he thought no more about; so when the enlargement first began to make its appearance he attributed it to a kick, never thinking of the former injury. The animal was taken home by the owner a few days after the operation, some distance in the country, and from last accounts was making an excellent recovery.

